# Simultaneous Contralateral Vestibular Schwannoma and Glomus Tumor of the Temporal Bone- A Case Report

**Published:** 2019-07

**Authors:** Hjalte-Christian-Reeberg Sass, Niels West, Jesper B. Yde

**Affiliations:** 1 *Department of Otolaryngology – Head and Neck Surgery and Audiology, Copenhagen University Hospital Rigshospitalet, Copenhagen*,* Denmark.*

**Keywords:** Acoustic neuroma, Audiology, Case Report, Glomus tumor, Vestibulogy

## Abstract

**Introduction:**

Presence of vestibular schwannoma and a simultaneous glomus jugulare tumor is an extremely rare event. There is only one case report regarding the incidence of a contralateral vestibular schwannoma, along with a glomus jugulare tumor. Herein, we present the second case with a contralateral tumor.

**Case Report:**

A 69-year-old woman presented with a long history of bilateral hearing loss and a 2-year history of left-sided pulsatile tinnitus. The patient also suffered the itching of the left ear canal and mild vertigo; however, she had no recollection of middle ear infection, ear discharge, or ear pain. Magnetic resonance imaging (MRI) revealed a right-sided 8-mm extrameatal vestibular schwannoma and a left-sided almost purely extracranial glomus jugulare tumor of 18 mm. The pure-tone average values were 63 and 43 dB HL for the right and left ears, respectively. Speech audiometry showed a discrimination score of 76/88 (%). Caloric irrigation was performed and revealed a unilateral weakness of 81% towards the side of vestibular schwannoma. The patient was included in a watchful waiting regimen with annual MRI scans.

**Conclusion:**

Though vestibular schwannomas and glomus jugulare tumors are pathophysiologically different, they are similar in terms of symptomatology, growth pattern, diagnostic process, and therapeutic strategy. Based on this case report, it can be concluded that a vestibular evaluation demonstrates a unilateral vestibular weakness towards the side of the vestibular schwannoma, thereby facilitating clinical discrimination between the lesions.

## Introduction

Vestibular schwannomas (VSs) originate from the Schwann cells of the 8^th^ cranial nerve. They account for around 80-90% of all cerebello- pontine angle tumors (1). Most of the VSs are sporadic and unilateral, with bilateral tumors being associated with neurofibromatosis type II (NF2) (2). Based on the statistics, almost 20 VSs per one million individuals are diagnosed each year (3).

Vestibular schwannomas present an enigmatic growth, which often occurs within the first five years. The large tumors may even remain stable through multiple years (4,5).

Glomus jugulare (GJ) tumors are slow-growing benign tumors, which can be locally aggressive. Such tumors originate from the paraganglionic cells in the adventitia of the jugulare bulb. The GJ tumors are rare with an annual incidence of 1 case per 1.3 million (5). Four cases of VS and simultaneous GJ tumor have been reported in the literature. Three of the four reported cases presented with ipsilateral VS and GJ tumors (6-8), while a single case had a VS and contralateral GJ tumor (9). Herein, we report a new case with contralateral tumors. However, the present case is unique since VS exhibited growth resulting in surgery while the patient was still in a watchful waiting regimen for the GJ tumor.

## Case Report

A 69-year-old woman presented with a long history of bilateral hearing loss and a 2-year history of left-sided pulsatile tinnitus. The patient also suffered from the itching of the left ear canal and mild vertigo; however, she had no recollection of middle ear infections, ear discharge, or ear pain. Otomicroscopy revealed the presence of a pulsatile purple-colored middle ear mass in the anterior inferior quadrant of the tympanic membrane of the left ear. The right ear had no visible abnormalities.

Due to the mild asymmetry in the pure-tone audiometry and the pulsatile tinnitus in the left ear, including the pulsating purple-colored mass, the patient was subjected to magnetic resonance imaging (MRI). 

**Fig1:**
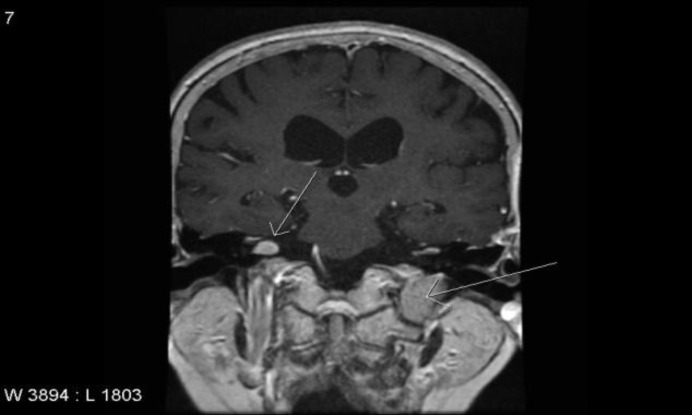
Coronal magnetic resonance imaging demonstrating a right-sided sporadic vestibular schwannoma (short arrow) and a left-sided glomus jugulare tumor (long arrow)

The MRI revealed a right-sided extrameatal vestibular schwannoma of 8 mm and a left-sided almost purely extracranial GJ tumor of 18 mm (Fig.1). After the diagnosis, the patient went into watchful waiting with annual follow-up MRI. 

**Fig 2:**
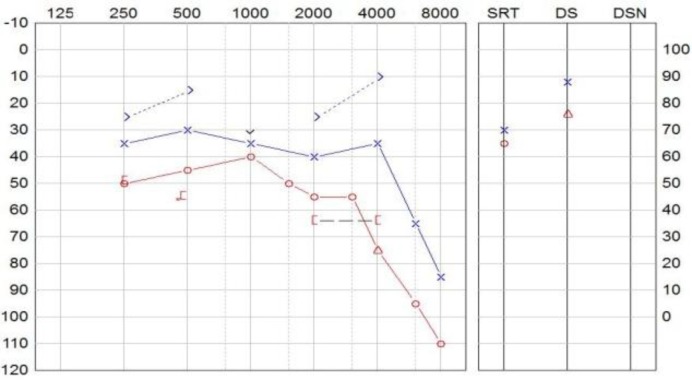
Audiogram showing bilateral hearing loss (Pure-tone audiometry suggests a right-sided sensorineural hearing loss and left-sided mixed hearing loss. The slightly better air conduction at 2 and 4 kHz must be attributed to the uncertainty of the test. Speech discrimination score is 76/88. Right-sided hearing acuity equivalents a class C hearing loss according to the American Academy of Otolaryngology-Head and Neck Surgery classification (10).

Two years after diagnosis, the patient experienced the deterioration of hearing and vertigo symptoms. The pure-tone audiometry (Fig.2) showed a bilateral though asymmetric hearing loss with a pure-tone average (PTA) of 63 and 43 dB HL on the right and left sides, respectively. Speech audiometry demonstrated a discrimination score of 76/88 (%). Caloric irrigation was performed and revealed a unilateral weakness of 81% towards the side of VS (Table.1). 

**Table 1:** Tumor characteristics and audiovestibular function of the vestibular schwannoma and glomus jugulare tumor

**Tumor**	**Side**	**Size**	**PTA**	**DS**	**Vestibular function**
Vestibular schwannoma	Right	12 mm	3	76	UW=81%
Glomus jugulare	Left	18 mm	43	88	Normal

PTA: pure-tone average, DS: discrimination score, UW: unilateral weakness

The patient was scanned independently of the standard MRI protocol. The MRI showed stationary dimensions of the GJ tumor; however, the extrameatal size of VS had increased from 8 mm to 12 mm. The patient underwent suboccipital retrosigmoid surgery with intended hearing preservation and the total removal of the VS.

## Discussion

Both VSs and GJ tumors are very rare benign neoplasms that commonly present with hearing loss. To the best of our knowledge, there is only one report regarding the incidence of VS, along with a contralateral GJ tumor (9). The patient presented binaural symptoms in the form of pulsatile tinnitus and hearing loss. The hearing was bilaterally affected in contrary to the previously published case. However, both PTA and discrimination scores were notably worse on the VS ear. In the present case, the caloric test could reveal a severe (81%) vestibular hypofunction ipsilateral to the VS. Vestibular affection is expected since VS arises from the vestibular branch of the 8^th^ cranial nerve ^4^. The MRI is the gold standard for the diagnosis of both VS and GJ tumors. 

Although the tumors are different in origin, they are similarly managed with observation, radiotherapy, or surgery. Yet, the formerly reported case was managed differently. In this regard, the GJ tumor in the mentioned case was treated with embolization and radiotherapy due to the tumor size, and the VS was subjected to a watchful waiting regimen. In our case, no GJ tumor growth was observed in the patient; on the contrary, she showed the growth of the vestibular schwannoma after 2 years, resulting in the need for active intervention. 

The patient chose surgery over radiotherapy despite the risk of hearing impairment. Postoperatively, the hearing was initially preserved; nonetheless, the patient experienced a continuous and gradual decrease in hearing on the operated ear. As a result, she was fitted with the contralateral routing of signal hearing aid with satisfactory results. The patient is still under a watchful waiting regimen for the GJ tumor. Based on the follow-up examinations, no growth or change has been observed in the tumor during the last 5 years. 

Despite the high pathophysiological difference between the VSs and GJ tumors, they have similar symptomatology, clinical presentation, growth pattern, diagnostic process, and treatment strategy. This case report is unique because it is the first attempt to accurately display the audiovestibular contradictions between a VS and a contralateral GJ tumor. Furthermore, no previously published cases of VS and left contralateral GJ tumor have been managed via a surgical approach and conservative regimen, respectively. 

## Conclusion

In this extremely rare case, the outcomes of the two tumors that differed in all aspects, except for site and dimensions, were determined by MRI. Though the hearing loss was bilateral, there was a noticeable asymmetry regarding the speech discrimination score indicating a retrocochlear affection on the VS side. In addition, the severe caloric areflexia signified a vestibular affection that in contrary to a GJ tumor could be explained by VS. 

Based on this case report, it can be concluded that a vestibular evaluation demonstrates a unilateral vestibular weakness towards the side of the VS, therefore facilitating clinical discrimination between the lesions. Studies investigating new methods to predict the characteristics of the tumors will greatly benefit the patients due to providing the ground for the implementation of a faster intervention, when necessary, and fewer follow-up scans.
